# Off label use of paliperidone LAIs in bipolar affective disorder with ADHD : from monthly to three monthly Trivicts

**DOI:** 10.1192/j.eurpsy.2026.11216

**Published:** 2026-07-31

**Authors:** O. S. Alkalbani

**Affiliations:** Psychiatry, Omsb, Muscat, Oman

## Abstract

**Introduction:**

Bipolar affective disorder (BAD) is a chronic psychiatric illness often complicated by treatment nonadherence, relapse, and comorbidities such as attention-deficit/hyperactivity disorder (ADHD). Long-acting injectable (LAI) antipsychotics are increasingly used off-label to address adherence, though evidence in BAD remains limited.

**Objectives:**

To describe the clinical course and outcomes of a patient with BAD and ADHD managed with paliperidone LAI, including transition from monthly (PP1M) to three-monthly (PP3M/Trivicta) formulation.

**Methods:**

We present the case of a 27-year-old Omani male with BAD and ADHD, followed from 2013 to 2025. Data were obtained from psychiatric records and serial follow-ups.

**Results:**

ADHD symptoms were first documented in adolescence (2013), characterized by impulsivity and academic decline. From 2015 onward, recurrent mood episodes led to multiple admissions, with poor adherence to oral risperidone and sodium valproate. In 2017, paliperidone palmitate LAI was initiated at family request, improving continuity but not fully preventing relapses. In December 2020, BAD was formally diagnosed following a manic episode. Between 2021–2022, he achieved partial stability on valproate and monthly LAI, but in 2023 transitioned to Trivicta (3-monthly) to address adherence challenges. Despite improved compliance, he was hospitalized in February 2025 with severe depression and suicidal ideation, requiring olanzapine augmentation, later switched to cariprazine. By August 2025, he remained euthymic, adherent to Trivicta, and functionally active.

**Conclusions:**

Paliperidone LAIs, including Trivicta, may enhance adherence and stabilization in BAD with comorbid ADHD, particularly in patients with repeated nonadherence. Nonetheless, depressive relapses may still occur, requiring adjunctive therapy. Further controlled studies are needed to clarify the role of LAIs in bipolar disorder

**Disclosure of Interest:**

None Declared

